# LIMPIC: a computational method for the separation of protein MALDI-TOF-MS signals from noise

**DOI:** 10.1186/1471-2105-8-101

**Published:** 2007-03-26

**Authors:** Dante Mantini, Francesca Petrucci, Damiana Pieragostino, Piero Del Boccio, Marta Di Nicola, Carmine Di Ilio, Giorgio Federici, Paolo Sacchetta, Silvia Comani, Andrea Urbani

**Affiliations:** 1 Istituto Tecnologie Avanzate Biomediche (ITAB), Fondazione "G. D'Annunzio", Chieti, Italy; 2 Centro Studi sull'Invecchiamento (Ce.S.I.), Fondazione "G.D'Annunzio", Chieti, Italy; 3 Dipartimento di Scienze Biomediche, Università "G. D'Annunzio", Chieti, Italy; 4 Centro Europeo Ricerca sul Cervello (CERC), IRCCS-Fondazione S. Lucia, Roma, Italy; 5 Dipartimento di Scienze Cliniche e Bioimmagini, Università "G. D'Annunzio", Chieti, Italy; 6 Dipartimento di Medicina Interna, Università di Roma "Tor Vergata", Roma, Italy; 7 Ospedale Pediatrico Bambino Gesù – IRCCS, Roma, Italy

## Abstract

**Background:**

Mass spectrometry protein profiling is a promising tool for biomarker discovery in clinical proteomics. However, the development of a reliable approach for the separation of protein signals from noise is required. In this paper, LIMPIC, a computational method for the detection of protein peaks from linear-mode MALDI-TOF data is proposed. LIMPIC is based on novel techniques for background noise reduction and baseline removal. Peak detection is performed considering the presence of a non-homogeneous noise level in the mass spectrum. A comparison of the peaks collected from multiple spectra is used to classify them on the basis of a detection rate parameter, and hence to separate the protein signals from other disturbances.

**Results:**

LIMPIC preprocessing proves to be superior than other classical preprocessing techniques, allowing for a reliable decomposition of the background noise and the baseline drift from the MALDI-TOF mass spectra. It provides lower coefficient of variation associated with the peak intensity, improving the reliability of the information that can be extracted from single spectra. Our results show that LIMPIC peak-picking is effective even in low protein concentration regimes. The analytical comparison with commercial and freeware peak-picking algorithms demonstrates its superior performances in terms of sensitivity and specificity, both on in-vitro purified protein samples and human plasma samples.

**Conclusion:**

The quantitative information on the peak intensity extracted with LIMPIC could be used for the recognition of significant protein profiles by means of advanced statistic tools: LIMPIC might be valuable in the perspective of biomarker discovery.

## Background

The research in protein biomarkers discovery is one of the fundamental topics in clinical proteomics in order to possibly improve both diagnosis and prognosis of a wide variety of disease states [1]. Mass spectrometry has proved to be the most promising tool in the perspective of biomarker identification [2,3]: it allows measuring the mass of ionized molecules, hence making it possible to analyze proteins in small concentrations and in a short time. In particular, excellent resolution and good mass accuracy combined with high sample throughput can be achieved with matrix-assisted laser desorption/ionization time-of-flight (MALDI-TOF) mass spectrometry [4,5]. The MALDI-TOF device produces signals that correspond to the different flight times of the analyzed proteins, which are ionized by means of a high energy laser beam and accelerated with an electric field: the ions, detected at the end of the tube, can be separated on the basis of their mass/charge ratio (*m/z*). The acquired spectra always present complex features, because the protein signals, characterized by "true" peaks in the mass spectrum, can be contaminated by several chemical and/or physical processes of the measurement procedure [6,7]. Two different kinds of disturbance can be revealed in the spectra: baseline drift and background noise. The baseline is the trend of the signal that would be generated by the mass spectrometer if no material was introduced into it. The background noise is a signal produced by electronic disturbances and fragments of material, with rapid fluctuations randomly varying over small mass ranges. As a consequence, a very sensitive and accurate peak-detection method, able to correctly separate "true" protein peaks from noise, is required [2]. Several methods using analytical rules, template matching and wavelet techniques have been proposed in the literature [6,8-11]. However, the problem of the detection of noise peaks as signals still remains a critical issue [12].

In this work, we propose a computational method for the detection of protein peaks from multiple MALDI-TOF-MS data, named LIMPIC (*linear MALDI-TOF-MS peak indication and classification*). Its major improvement is a new procedure for decomposing a MALDI-TOF mass spectrum into signal, baseline and noise. Subsequently, LIMPIC estimates an non-uniform residual noise level from processed spectra, and it detects protein signals by finding peaks that have significantly high signal-to-noise ratio. The peak lists generated from the single spectra are then compared, and a classification between molecular signal peaks and noise is performed on the basis of a detection rate parameter. In-vitro purified protein samples and human plasma samples were used for the validation of LIMPIC: in both conditions, the proposed method showed a significant accuracy in the detection of the protein signals, and it was able to provide a superior sensitivity and specificity than two well-established commercial algorithms, APEX and CENTROID, and a freely available algorithm, CROMWELL.

## Results

**Figure 1:**
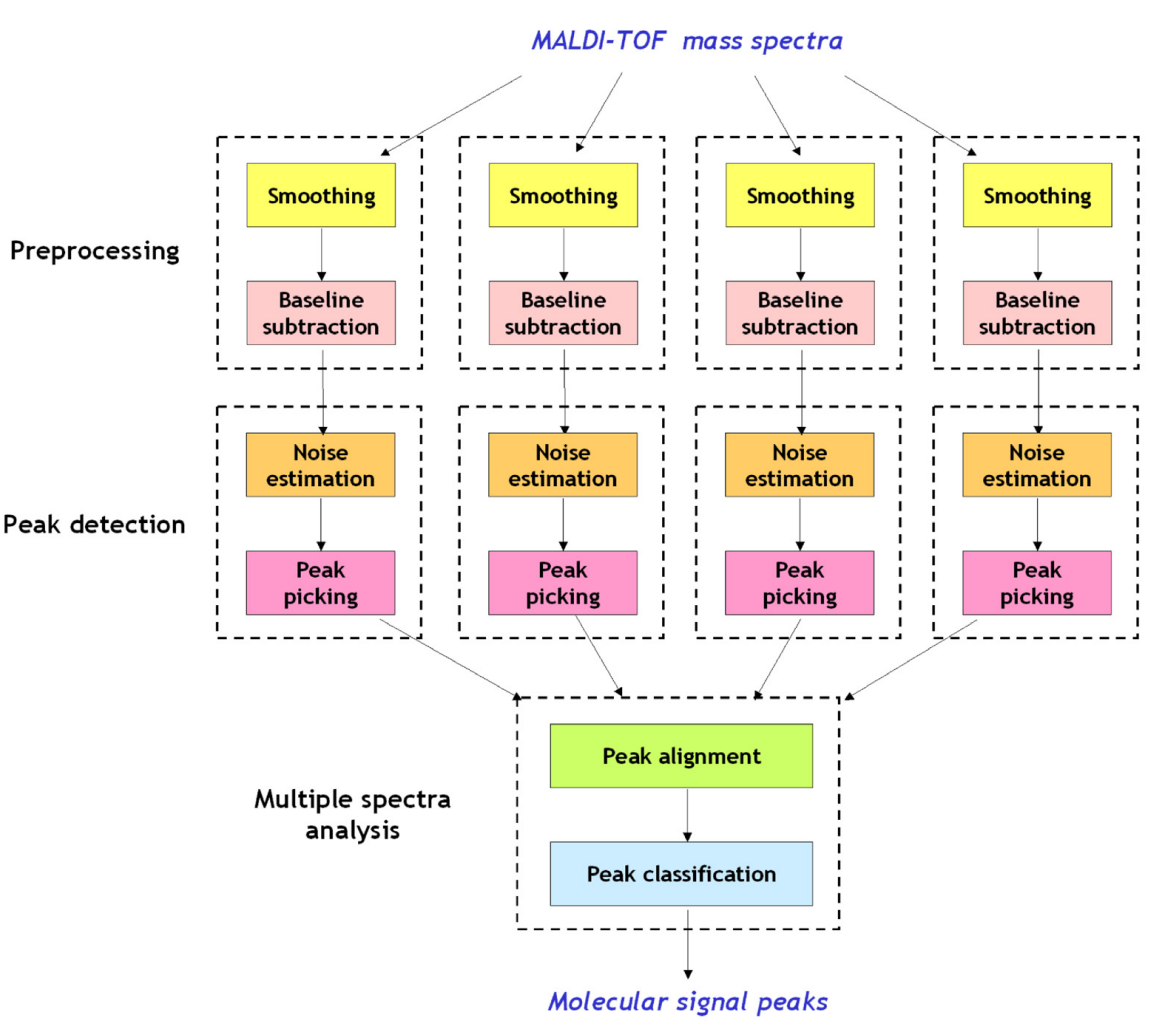
**LIMPIC architecture**. Schematic representation of the LIMPIC software. Through its processing and analysis steps, LIMPIC retrieves information from a set of calibrated MALDI-TOF mass spectra, and provides a list of "true" molecular signal peaks.

The LIMPIC method was developed for the detection of consistent protein peaks, starting from a set of calibrated mass spectra. It was implemented in MATLAB, and is provided as Additional File 1. A schematic overview of its processing and analysis steps is given in Figure 1. Each mass spectrum is preprocessed with smoothing and baseline removal. The smoothing is performed by means of a Kaiser digital filter working with a moving window [13]; the baseline removal is accomplished with the subtraction of a signal trend, estimated after the elimination of the most significant peaks in the mass spectrum. The detection of protein peaks in the single spectra is performed by finding all the local maxima, and eliminating those with intensity lower than a non-uniform threshold, proportional to the noise level [14]. The peaks detected for the single spectra are clustered on the basis of their m/z position, and then classified as protein or noise peaks on the basis of their consistency across the spectra [15,16].

The validation of the proposed method for the separation of MALDI-TOF-MS signals from noise regarded two aspects: the effectiveness of signal processing and the detection of reliable protein peaks. For this purpose, we used mass spectra from in-vitro purified protein mixtures for testing LIMPIC under controlled conditions, and human plasma samples for validating it in the perspective of clinical applications. The two sets of MALDI-TOF mass spectra are respectively provided as Additional Files 2 and 3. In this study, we also compared the outcomes of LIMPIC with those of two commercial algorithms, APEX and CENTROID (implemented in the software package FlexAnalysis by Bruker Daltonics) [17,18], and of a recently released freeware algorithm, CROMWELL, created by the Texas University bioinformatics group [10,19].

**Figure 2:**
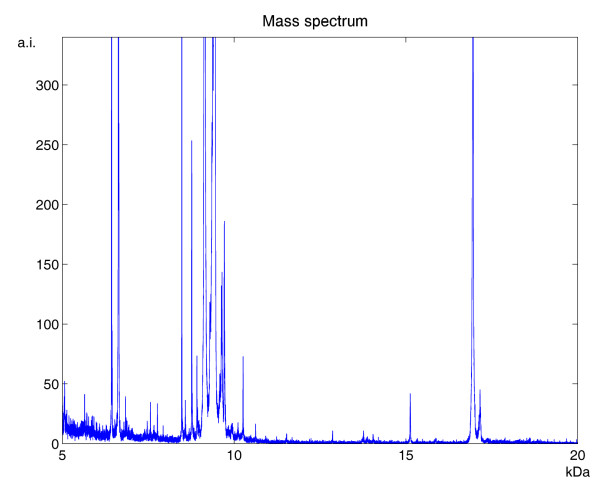
**MALDI-TOF-MS spectrum from a human plasma sample**. Example of raw MALDI-TOF mass spectrum acquired from human plasma, showing the presence of background noise and baseline drift.

**Figure 3:**
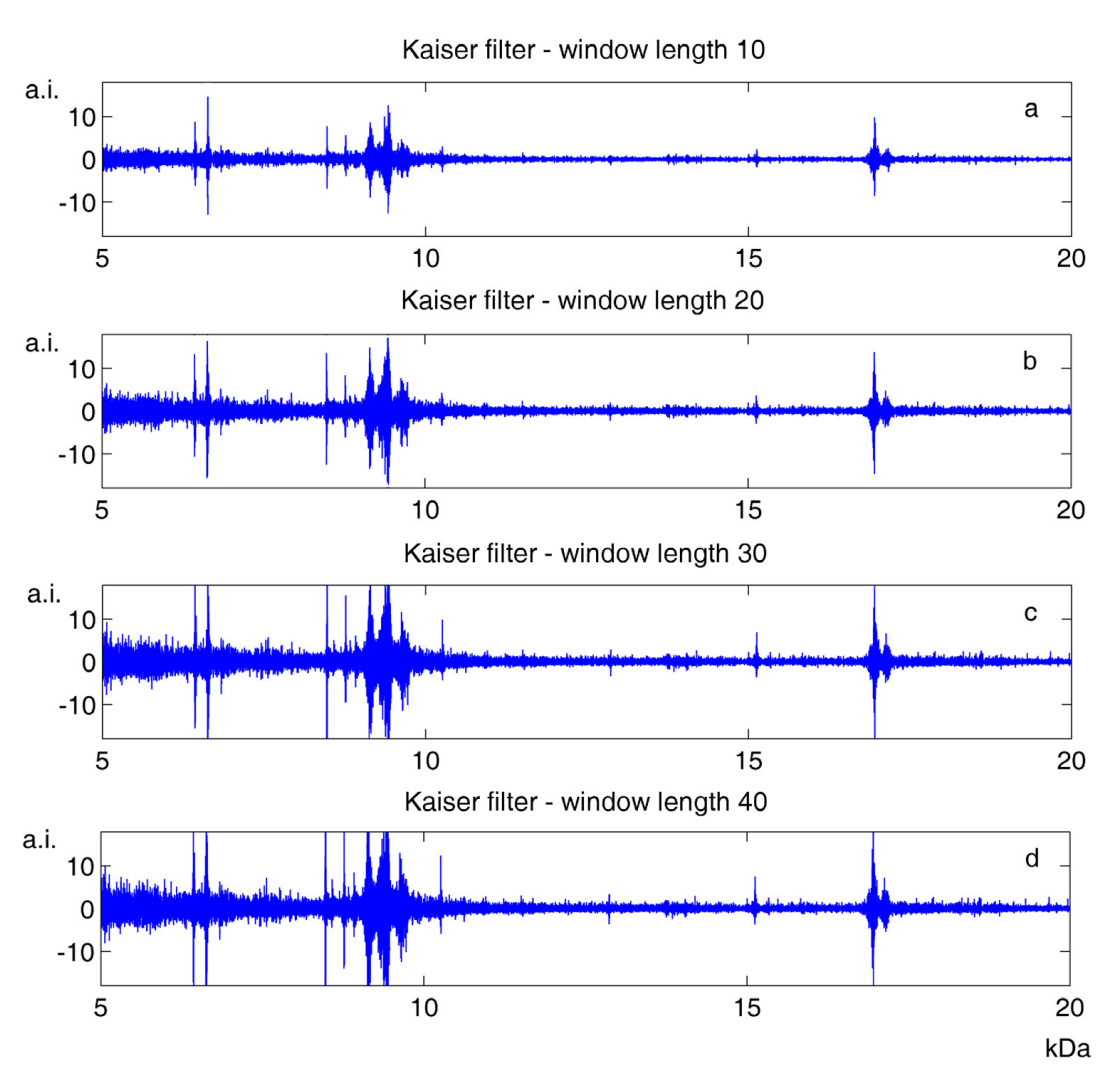
**Analysis of Kaiser filter denoising**. The Kaiser filter performances can be appreciated from the difference between mass spectra before and after smoothing. The outcomes for the mass spectrum shown in Figure 2, referring to moving window length equal to 10, 20, 30 and 40 data points, are respectively presented in (a), (b), (c) and (d).

**Figure 4:**
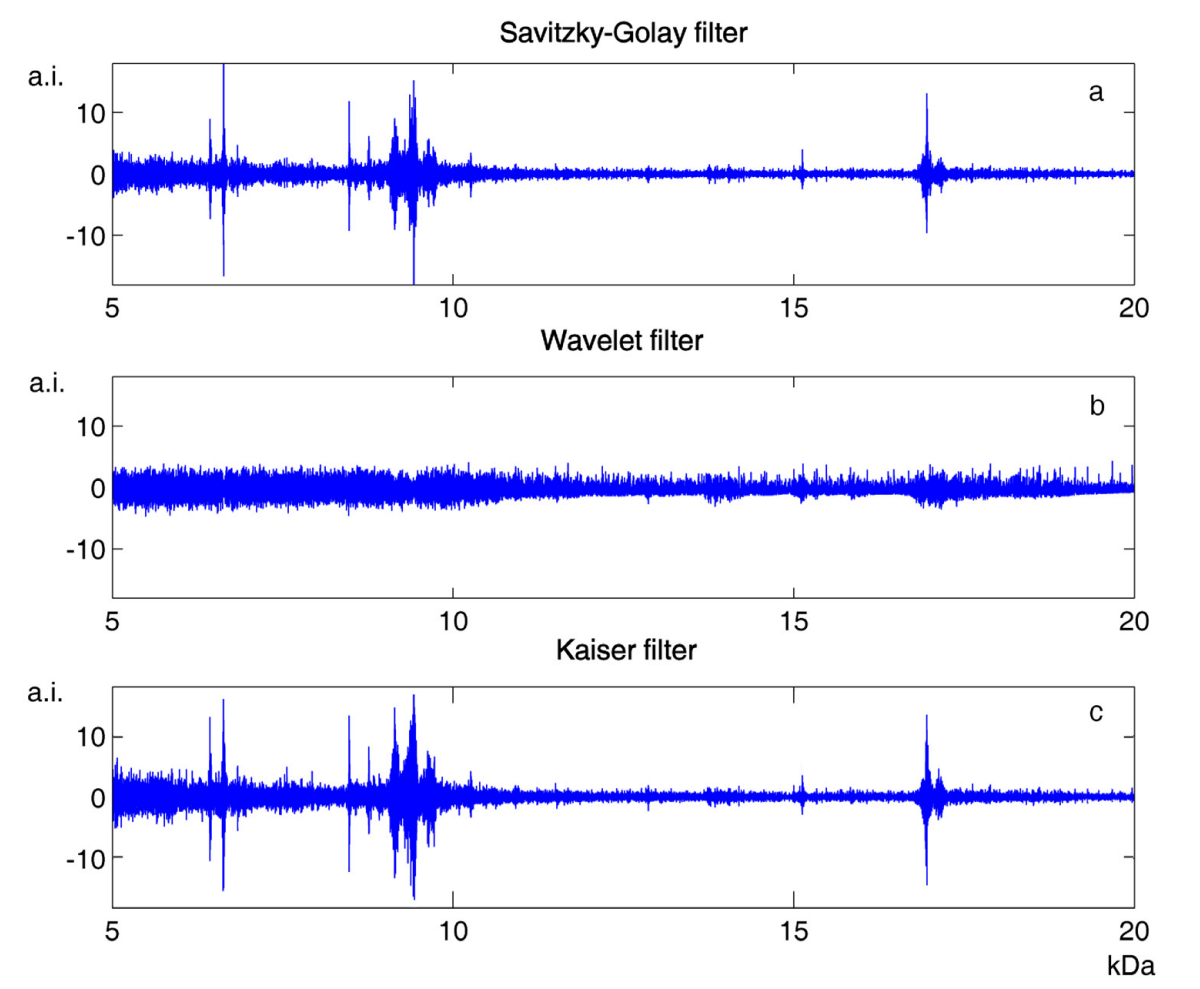
**Smoothing performance comparison**. The performances of several smoothing algorithms can be appreciated from the difference between mass spectra before and after smoothing. The outcomes obtained from Savitzky-Golay, Wavelet and Kaiser filters for the mass spectrum shown in Figure 2 are respectively illustrated in (a), (b) and (c).

An example of MALDI-TOF data from plasma samples, noticeably contaminated by baseline drift and background noise, is illustrated in Figure 2. With regard to spectrum denoising, we analyzed the performances of Kaiser filter with different window lengths, in order to find the general criteria for setting this parameter. In fact, small window lengths might result in an insufficient denoising, whereas large ones might distort the "true" signal. As shown in Figure 3, we explored 4 different parameter settings, with window length ranging between 10 and 40 data points. Analyzing the subtracted noise, we found that its amplitude increased with the window length; however, we observed that the smoothing using the two larger window lengths produced signal distortions, evidenced by a peaked signal distribution exactly where peaks occurred in the mass spectrum. Consequently, we decided to set this parameter equal to 20, thus covering a range of 5 Da in the mass spectrum. The performances of Kaiser filter in terms of data smoothing were compared with those of Savitzky-Golay filter [20], the solution generally adopted in FlexAnalysis with APEX and CENTROID, and Wavelet filter [10], the smoothing technique used in CROMWELL. For Savitzky-Golay filter, that is also based on a moving window, we used the same window length as for Kaiser filter, whereas for Wavelet filter we adopted the parameters suggested by the CROMWELL research group. As illustrated in Figure 4, Wavelet filter provides very different outcomes with respect to the two moving-window filters: it was minimally influenced by the presence of peaks in the mass spectrum, but it did not seem to properly reconstruct noise structure at the high end of the studied mass range. We measured the similarity of the noise structures with respect to Gaussian noise by means of two statistical parameters: skewness and kurtosis. Surprisingly, Wavelet filters presented a significant value of skewness, that is related to the presence of an asymmetrical noise distribution. Conversely, Savitzky-Golay and Kaiser filters showed noise structures consistent with respect to the mass spectrum, and more similar to Gaussian noise, with relatively low values of skewness and kurtosis.

**Figure 5:**
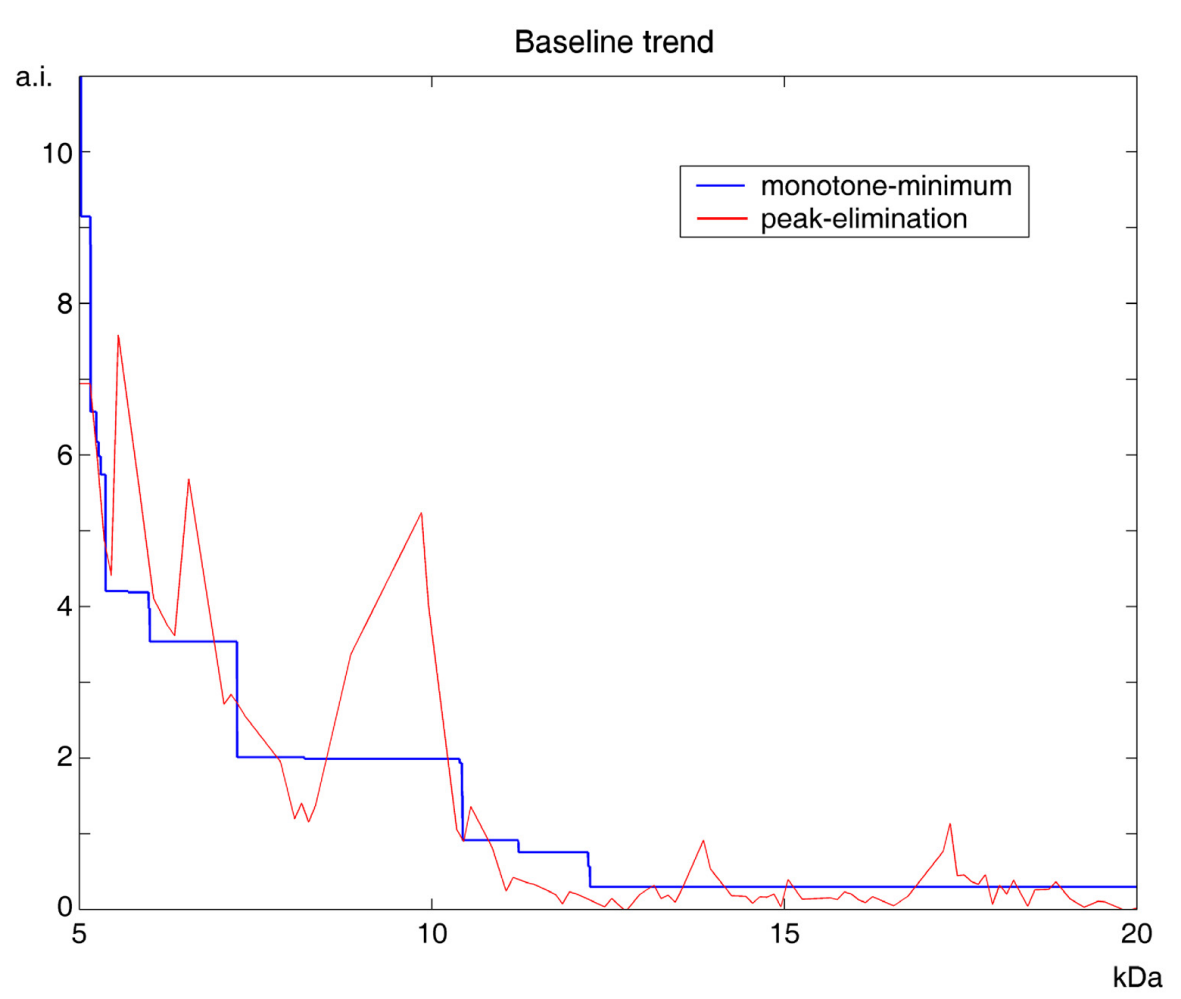
**Baseline removal performance comparison**. The baseline drift estimated with the minimum-value interpolation method (APEX, CENTROID and CROMWELL processing) is compared with that of the peak-elimination method (LIMPIC processing).

With regard to baseline removal, we compared the peak-elimination method used in LIMPIC with the classical method based on minimum value interpolation [6,10], used by APEX, CENTROID and CROMWELL. The two estimates of the baseline drift are superimposed in Figure 5. The most evident difference is that the LIMPIC baseline can locally increase, for example when large and overlapping peaks are present in the spectrum.

**Figure 6:**
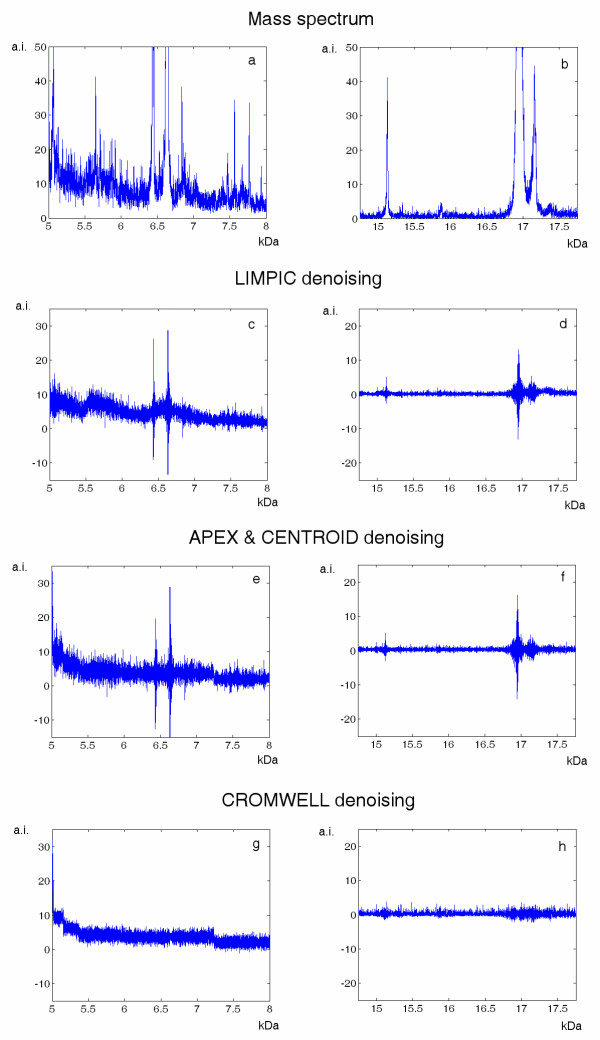
**Denoising performances**. The MALDI-TOF mass spectrum presented in Figure 2 is shown in the mass ranges 5–8 kDa (a) and 14.75–17.75 kDa (b). The comparison between the noise subtracted by LIMPIC (c-d), APEX and CENTROID (e-f), and CROMWELL (g-h) in the same mass ranges is presented in the panes below.

A qualitative comparison referring to the mass spectrum shown in Figure 2, before and after smoothing and baseline subtraction, is provided in Figure 6 for LIMPIC, APEX, CENTROID and CROMWELL processing. Furthermore, the reliability of intensity reconstruction was assessed using mass spectra acquired from in-vitro purified protein mixtures of equine myoglobin and cytochrome C. This analysis was carried out at 6 different concentrations of cytochrome C, ranging between 2.5 and 50 fmol. For each concentration, the coefficient of variation was calculated on 5 replicate spectra for the peak corresponding to the single-protonated molecular ion of cytochrome C (Table 1). LIMPIC processing provided results that were comparable to APEX, CENTROID and CROMWELL above 10 fmol, whereas it allowed a more reliable estimate of signal intensity at lower protein concentrations.

**Table 1:** Performance comparison of preprocessing methods.

*Protein concentration*		*2.5 fmol*	*5 fmol*	*7.5 fmol*	*10 fmol*	*25 fmol*	*50 fmol*
*LIMPIC*	Average intensity	1.17	4.65	5.32	17.07	94.03	277.37
	Standard deviation	0.10	0.39	0.61	2.70	14.01	42.48
	Coefficient of variation	0.09	0.08	0.12	0.16	0.15	0.15

*APEX & CENTROID*	Average intensity	1.31	5.36	6.05	14.94	88.48	283.08
	Standard deviation	0.32	0.74	0.97	2.40	12.76	42.48
	Coefficient of variation	0.24	0.14	0.16	0.16>	0.14	0.15

*CROMWELL*	Average intensity	0	0	4.01	12.76	85.34	275.50
	Standard deviation	0	0	1.52	2.95	13.79	48.49
	Coefficient of variation	-	-	0.38	0.23	0.16	0.17

LIMPIC, APEX, CENTROID and CROMWELL preprocessing methods were evaluated from in-vitro protein spectra at different dilutions of cytochrome C. For each method, the average peak intensity, the standard deviation and the coefficient of variation are provided. The underlined values were manually obtained from the mass spectra, because the peak-picking algorithms did not allow to detect the peak.

In particular, CROMWELL produced null intensity for the cytochrome C peak at 2.5 fmol, including it in the subtracted noise, and APEX and CENTROID showed a coefficient of variation equal to 24%, which might be ascribed to a residual noise level comparable to the peak intensity. In this case, the coefficient of variation after LIMPIC processing was equal to 8%.

**Figure 7:**
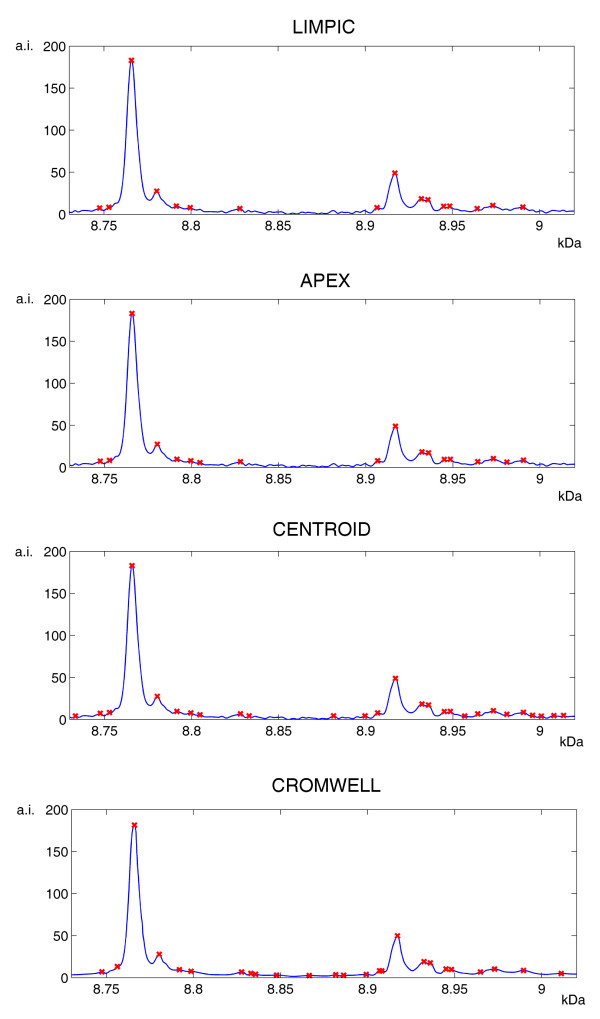
**Peak-picking for a single mass spectrum**. Example of the peak-detection results for the four algorithms. For each of them, a plot of the mass spectrum shown in Figure 2 in the mass range 8.73–9.02 kDa is shown, along with the detected peaks, marked by red crosses.

**Figure 8:**
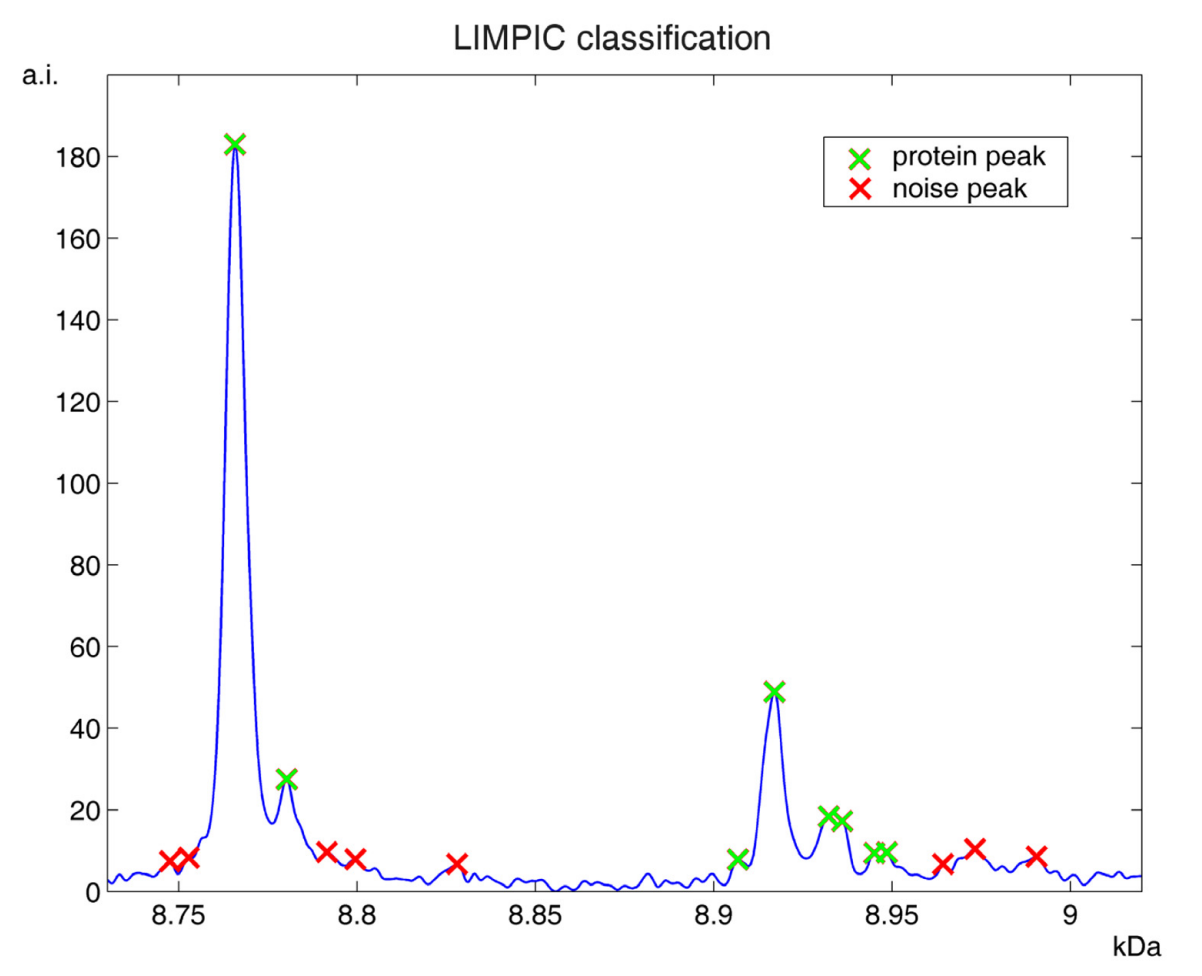
**LIMPIC classification of protein and noise peaks**. The same peaks detected by LIMPIC, shown in figure 7, classified after multiple-spectra comparison. The peaks with detection rate across spectra larger than or equal to 0.5, being considered protein peaks, are marked with green crosses, whereas the remaining peaks are assumed to be ascribed to noise and are marked with red crosses.

Using the same in-vitro protein mass spectra, the peak detection sensitivity of LIMPIC was compared with that of APEX, CENTROID and CROMWELL. The latter does not need parameter tuning; conversely, a number of input parameters are required by the two commercial algorithms. Therefore, we adopted the typical parameters used in our lab: SNR = 3 and peak width 0.75 Da for APEX; SNR = 3, peak width 0.75 Da and percent height 80% for CENTROID. In order to have comparable results, the peaks detected with the commercial algorithms and with CROMWELL from the replicate spectra were clustered as for LIMPIC. We also classified the peaks on the basis of a parameter named *peak detection rate *(PDR), expressed by the ratio between the number of spectra containing the considered peak and the total number of analyzed spectra. As shown in detail in Table 1, only LIMPIC was capable to reveal the cytochrome C peak in case of low protein concentration regimes (< 10 fmol), when ionic competition processes in the mass spectra produced a loss of linearity between the protein abundance and the related peak intensity. The comparative results of the peak-picking algorithms for all peaks are summarized in Table 2. The correct identification of the protein peaks was generally accomplished with LIMPIC for all the acquired signals, with no false negatives and only one false positive among the 54 detected peaks. On the other hand, a large number of incorrect peaks was produced by APEX, CENTROID and CROMWELL. These results were confirmed by those obtained with plasma spectra, for which an example of the peaks detected by the four methods from a single spectrum is provided in Figure 7. When comparing the peaks from the single spectrum with those obtained from the other plasma spectra, we found that only a part of them is consistent. In Figure 8 we provide an example of the LIMPIC classification, performed for the same peaks shown in Figure 7, using a minimum PDR equal to 0.5.

**Table 2:** Performance comparison of peak-detection algorithms.

*Protein concentration*		*2.5 fmol*	*5 fmol*	*7.5 fmol*	*10 fmol*	*25 fmol*	*50 fmol*
Number of manually detected peak classes		7	7	10	8	10	11

*LIMPIC*	Number of detected classes	8	7	10	8	10	11
	Number of missed classes	0	0	0	0	0	0
	Number of incorrect classes	1	0	0	0	0	0

*APEX*	Number of detected classes	27	18	34	24	25	21
	Number of missed classes	1	1	3	1	0	2
	Number of incorrect classes	21	12	27	17	15	12

*CENTROID*	Number of detected classes	40	52	48	24	25	27
	Number of missed classes	1	1	1	0	0	0
	Number of incorrect classes	34	46	39	16	15	16

*CROMWELL*	Number of detected classes	14	15	14	16	21	24
	Number of missed classes	3	3	6	2	3	4
	Number of incorrect classes	10	11	10	10	14	17

LIMPIC, APEX, CENTROID and CROMWELL methods were assessed from in-vitro protein spectra at different cytochrome C dilutions, with respect to the number of detected peak classes, false negatives and false positives. Only the peak classes that could be found across replicate spectra with a peak detection rate (PDR) larger than 0.5 were considered for the analysis.

**Figure 9:**
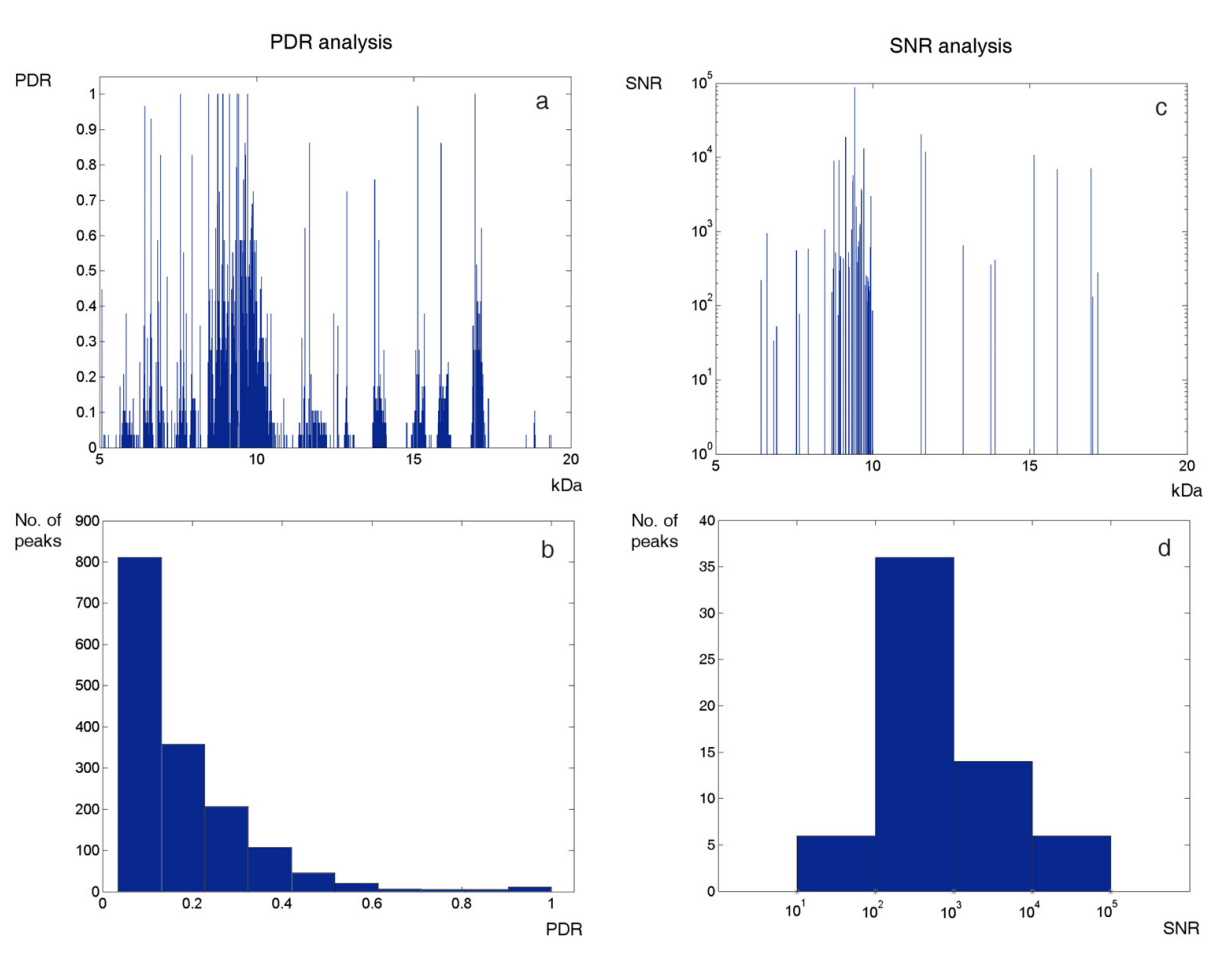
**Multiple-spectra analysis of the peaks detected with LIMPIC**. The results of peak detection rate (PDR) associated with all the LIMPIC peak classes are presented for plasma mass spectra, as well as those of signal-to-noise ratio (SNR) of the "true" protein peaks, characterized by a PDR larger than or equal to 0.5. (a) For each peak class, the PDR is represented in linear scale as a vertical line, which is positioned in correspondence of the related *m/z *value; (b) Histogram of the PDR values shown in (a); (c) For each selected peak class, the SNR is represented in logarithmic scale as a vertical line, which is positioned in correspondence of the related *m/z *value; (d) Histogram of the average SNR values shown in (c).

Analytical results about peak masses and peak intensities for the group of plasma samples are summarized in a spreadsheet, which has been provided as Additional File 4. We grouped the 4382 peaks detected among all the MALDI-TOF-MS signals into 1581 peak classes. This allowed selecting the most reliable peaks, because only the ones that are robustly found across spectra were retained. Using a minimum PDR equal to 0.5, 62 out of the 1581 peak classes were selected (Figure 9). In order to determine whether the selected peaks could be used for quantitative studies, the SNR values were analyzed. In particular, the condition SNR > 10 was fulfilled for all the 62 peaks, as illustrated in Figure 9.

Using the selection criterion based on the PDR value, it was also possible to measure the *hit-rate*, defined as the ratio between the number of "true" peak classes and the average number of the peaks revealed in the single spectra: the larger this ratio the better the performances of the peak-picking method. According to this parameter, a larger number of consistent peaks were generally detected with LIMPIC, confirming the superior reliability of this method for mass spectra from both protein mixtures and plasma samples (Table 3).

**Table 3:** Hit-rate of peak-detection algorithms.

		*LIMPIC*	*APEX*	*CENTROID*	CROMWELL
*In-vitro proteins*	Average number of detected peaks	16	42	62	50
	Number of peak classes	11	21	27	23
	Hit-rate	0.69	0.50	0.44	0.44

*Human plasma*	Average number of detected peaks	146	291	283	172
	Number of peak classes	62	84	76	60
	Hit-rate	0.42	0.29	0.27	0.35

LIMPIC, APEX, CENTROID and CROMWELL methods were assessed with respect to the groups of mass spectra obtained from the in-vitro purified protein samples and human plasma samples, respectively. The performances were measured on the basis of a *hit-rate *parameter; the latter was obtained as a ratio between the number of peak classes, selected on the basis of a PDR larger than 0.5, and the average number of detected peaks.

**Figure 10:**
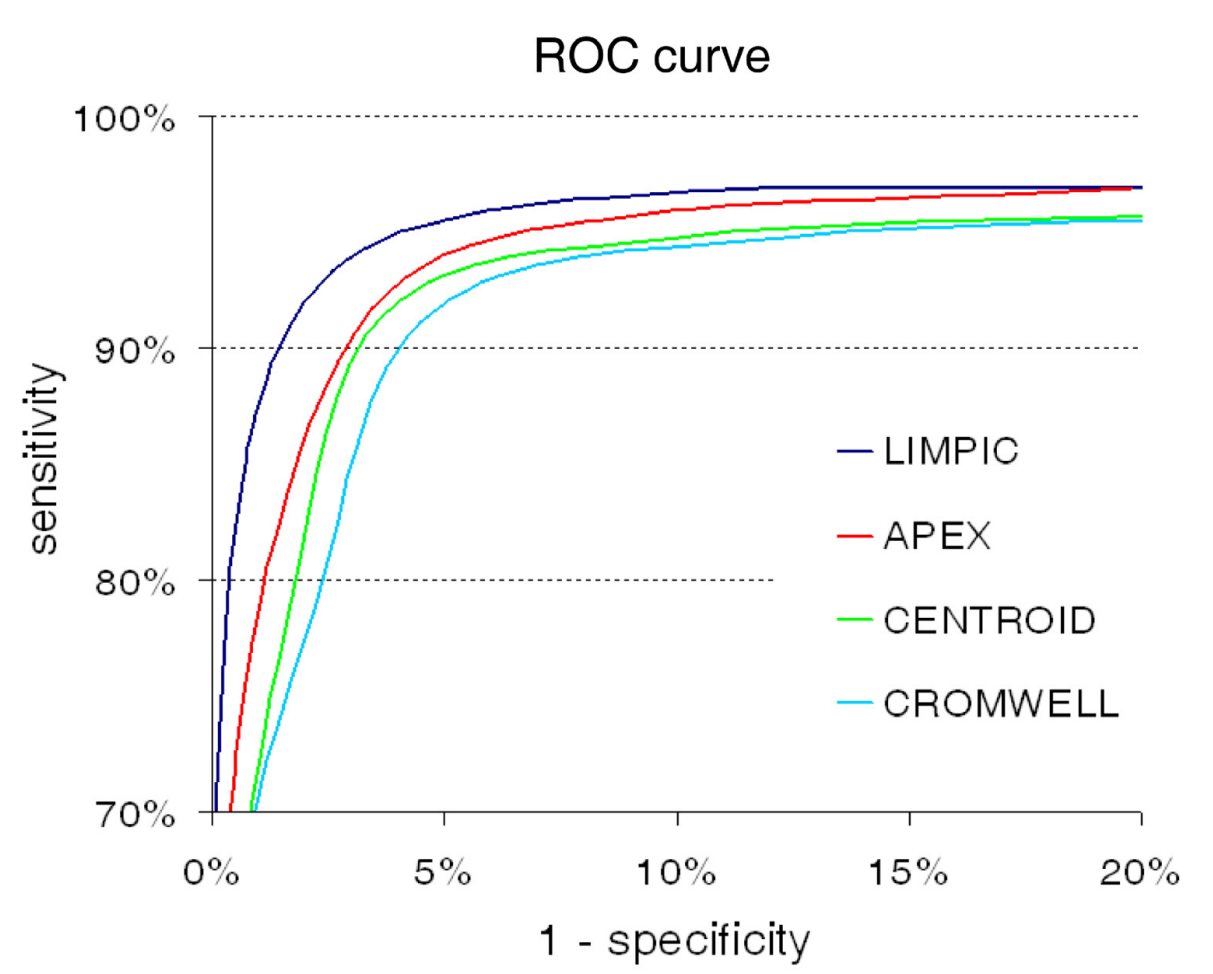
**ROC curves calculated for peak-detection methods with human plasma samples**. ROC curves showing the sensitivity and specificity of LIMPIC, APEX, CENTROID and CROMWELL. These indexes have been computed for the mass spectra obtained from human plasma.

Moreover, with regard to the classification of peaks from plasma mass spectra, we analyzed the PDR distribution, in order to obtain valuable information regarding the method sensitivity in the separation of protein and noise peaks. As it was impossible to accurately define the presence of a defined set of protein peaks, the detected peaks were compared with a given list of 68 peaks that correspond to proteins classified in the Human Plasma Proteome Project (HPPP) database [21] which can be revealed by MALDI-TOF mass spectrometry in the *m/z *range 5–20 kDa [22]. The complete list of the proteins considered for this analysis can be found in Additional File 5. A ROC curve was calculated for LIMPIC, APEX, CENTROID and CROMWELL, varying the minimum PDR used for the peak classification (Figure 10). The results demonstrated that a larger accuracy can be achieved with LIMPIC; in particular, no false positives were obtained by setting minimum PDR to 0.6, whereas the best performances in terms of sensitivity and specificity were attained by setting minimum PDR to 0.4.

## Discussion

We presented a reliable automated tool for signal denoising and peak detection in MALDI-TOF-MS data. In contrast to many established approaches to this problem [6,8-11], the proposed method was particularly designed to work properly even on very low intensity peaks. LIMPIC processing reduced the disturbances that partially hid the true signal in the mass spectrum. In particular, the smoothing algorithm permitted the reduction of the background noise without any noticeable distortion of the true signal, and it seemed to be more effective than Savitzky-Golay and Wavelet filters with MALDI-TOF-MS data. Moreover, the LIMPIC baseline correction was able to work with all kinds of spectra, even with those characterized by a non-increasing baseline drift, for which the standard method based on fitting a monotone minimum curve might be unsuccessful [10]. In addition, the coefficient of variation obtained with LIMPIC with in-vitro protein mixtures at low concentrations proved the efficacy of the preprocessing steps, and also the reliability of the quantitative information that could be extracted from single peaks. According to the method proposed by Yasui [8], the positions of the peaks in the mass spectrum were determined on the basis of the first derivative of the signal, whereas other techniques based on template matching were considered unreliable, because the presence of residual noise could corrupt the ideal peak shape and could determine incorrect outcomes [23].

Given the significant consistency of noise level across spectra, also after preprocessing, an alternative technique for the reduction of false peak discovery was proposed in the literature [15]. It is not based on data smoothing, but on the determination of a proper non-uniform threshold, in order to exclude intensities associated with the noise. The noise level is calculated by means of the local standard deviation, considering data points with intensity below the 90^th ^percentile. As a result, this approach might be inappropriate in case of spectra with a high number of large-intensity peaks. Also our method takes into account the presence of an uneven noise level, but in our opinion it is suitable for a broader variety of mass spectra: it estimates a non-uniform noise threshold on the basis of the waveform properties, without making any assumptions on the signal intensities.

Although the IUPAC guidelines suggest the use of the condition s > 10σ to extract quantitative information on signal intensity [14], we found that the LIMPIC configuration with s > 3σ still yields good performances, allowing an accurate identification of weak peaks. However, in light of the findings of other research groups using different methods, it is clear that the number of peaks detected in the single spectrum is not, by itself, a sufficient measure of the peak-picking effectiveness [10]. Since we matched the results obtained from replicate spectra, we could also appraise the amount of peaks that, after the comparison, were likely to be classified as noise peaks [16]. The PDR analysis was aimed at obtaining an optimal separation between protein signals from noise. In particular, the optimal cut-off value had to be properly chosen, depending on the specific application: the selection of a large value (e.g. PDR ≥ 0.6) increased the reliability of the selected peaks, but simultaneously reduced the amount of information that can be used for subsequent analyses. When a relatively small value is chosen (e.g. PDR ≥ 0.4), the probability of selecting "noise" peaks can be reduced by also setting a threshold for the minimum acceptable SNR. Nonetheless, the selection of peaks with SNR > 10 allows, in the perspective of biomarker discovery, to categorize the ones for which the associated intensity values can be used for statistical tests.

Although the number of the detected peaks was generally smaller for LIMPIC than for the commercial algorithms APEX and CENTROID, and for the freeware algorithm CROMWELL, the larger hit-rates of the proposed method demonstrated that a more reliable selection of real protein signals can be accomplished. Therefore, the ROC curves calculated for the four methods reveal that a superior sensitivity and specificity can be obtained with LIMPIC, particularly when a low cut-off value was used for the PDR.

## Conclusion

The discovery of protein profiles that can be ascribed to each pathologic state and the classification of individual proteins that compose them are key steps in the direction of an early disease detection. The proposed method was intended for the reliable detection of protein peaks using average mass signals such as the one collected from linear MALDI-TOF-MS instruments. In this case, data processing is particularly difficult, because weak protein signals, represented by the "true" peaks in the acquired spectra, are generally hidden by two disturbances: baseline drift and background noise. We developed a novel technique for mass spectrometric data denoising, and we showed that the processed spectra can be jointly used for peak detection with high reliability and accuracy. The LIMPIC method was properly designed in order to work correctly also in case of very weak peaks. Moreover, the entire processing procedures and algorithms can be modified and tailored to the data produced by different acquisition systems, allowing for a complete control, which is rarely possible in commercial software solutions. With the recent improvements in the MALDI-TOF technology, the detection of protein peaks with a low coefficient of variation across measurements is becoming feasible; as a consequence, the quantitative information on the peak intensity extracted with our method could be used for the recognition of significant protein profiles by means of advanced statistic tools. We hope that the analytic strategy presented here using mass spectrometric data will support further advances for the discovery of novel disease-state biomarkers.

## Methods

### MALDI-TOF-MS analysis

The MALDI-TOF mass spectra used in this study were acquired from both human plasma samples and in-vitro protein samples: the former were used to validate it in the perspective of clinical applications, and the latter were used to test the proposed method under controlled conditions.

The human plasma samples were collected from 30 healthy subjects (age 28–40 years) who signed an informed consent approved by the Local Ethical Committee. Equine myoglobin was dissolved in 0.1% trifluoroacetic acid (TFA) in deionized water and was used as calibrator. Plasma sample preparation was performed using ZipTip (Millipore) C4 tips with sinapinic acid [24]. The plasma samples (20 μl) were first acidified by addition of 5 μl 1% TFA before loading and preparation with a sandwich layer method on MTP ground steel 384 (Bruker Daltonics). First, a sinapinic acid matrix seed layer was created by depositing a droplet (0.5 μl) of a saturated solution of sinapinic acid in 100% ethanol on the target. The C4 resin was first activated by multiple washing with 10 μl of ACN/water (1:1) and then equilibrated by 0.1% TFA. Thereafter the sample was trapped on the ZipTip resin and washed with TFA 0.1%; finally the sample was eluted from the resin using 2 μl of a saturated solution of sinapinic acid in 30/70 ACN/0.1% TFA and spotted directly on MTP ground steel 384 (Bruker Daltonics).

The in-vitro protein samples were prepared at 6 different dilutions, combining equine myoglobin and cytochrome C. The first protein was maintained at the fixed concentration of 0.5 pmol/μl, whereas the concentration of the second one was progressively reduced from 50 fmol/μl to 2.5 fmol/μl. First, 1 μl of each sample was acidified by the addition of 9 μl 0.1% TFA. The resulting solution was manually spotted with a ground steel onto 5 different matrix droplets of the MALDI target using the sinapinic acid sandwich method [24].

All MALDI analyses were performed with a MALDI-TOF mass spectrometer Bruker-Daltonics Reflex IV, equipped with a nitrogen laser (337 nm) [5]. The ion source and flight tube were evacuated by turbo pumps to a pressure lower than 6 × 10^-7 ^mbar. All spectra were acquired in linear mode for a mass range of 5–20 kDa, at a voltage of 20, 17 and 9.60 kV for the first and second ion extraction stage and lens, respectively. The laser power was modulated between 20% and 40% in order to obtain less than 5 × 10^2 ^ion counts for a single acquisition run. Every single acquisition run was composed by 100 laser pulses at 5 Hz; multiple additions of single position acquisition run were employed to obtain a minimal spectrum intensity scale of 5 × 10^3 ^ion counts. The m/z resolution of the resulting mass spectra was decreasing from 0.33 to 0.70 Da from the low end to the high end, respectively, of the considered mass range. In addition, peak resolution, based upon the width of the peak at half the maximum intensity (FWHM), was always larger than 1100 at the low end and than 900 at the high end of the mass range.

### Data processing and peak detection

To circumvent the problem of *m/z *shifts among spectra, the signals were calibrated using the two main peaks derived from myoglobin ([M + H]^+^and [M + 2H]^2+ ^ions of myoglobin at *m/z *16952 and 8476, respectively) as internal calibrants [7,25]. The spectra were also normalized using the main peak intensity of the same protein. In order to be suitable for data preprocessing, each linear-mode MALDI-MS spectrum was converted to a text file listing of 6 × 10^4 ^intensities versus *m/z *data points, spaced 0.25 Da from each other.

The classical approach for MALDI-TOF-MS data processing consists in decomposing the acquired mass spectrum *r *into three components: the true signal *s*, the baseline drift *c*, and the environmental noise *σ*. Consequently, each mass spectrum can be schematically modeled by the equation

*r *= *s *+ *c *+ *σ *    (1)

The true signal consists of a number of peaks at different *m/z *values, the intensity of which of the same order of magnitude as the background noise in same cases [6]. Consequently, a noise reduction technique was performed at first: the signal-to-noise ratio (SNR) was enhanced by using a smoothing procedure based on a Kaiser filter [13]. In short, a Kaiser filter is a Finite-Impulse-Response (FIR) filter that approximates an ideal low-pass filter, while attempting to minimize the ripples in the frequency response caused by the signal truncation.

Given a set of input values *w*(*t*), a generic FIR filter generates an impulse response *p*(*t*) of the following form:

p(t)=∑n=0lanw(t−n)     (2)
 MathType@MTEF@5@5@+=feaafiart1ev1aaatCvAUfKttLearuWrP9MDH5MBPbIqV92AaeXatLxBI9gBaebbnrfifHhDYfgasaacH8akY=wiFfYdH8Gipec8Eeeu0xXdbba9frFj0=OqFfea0dXdd9vqai=hGuQ8kuc9pgc9s8qqaq=dirpe0xb9q8qiLsFr0=vr0=vr0dc8meaabaqaciaacaGaaeqabaqabeGadaaakeaacqWGWbaCcqGGOaakcqWG0baDcqGGPaqkcqGH9aqpdaaeWbqaaiabdggaHnaaBaaaleaacqWGUbGBaeqaaOGaem4DaCNaeiikaGIaemiDaqNaeyOeI0IaemOBa4MaeiykaKcaleaacqWGUbGBcqGH9aqpcqaIWaamaeaacqWGSbaBa0GaeyyeIuoakiaaxMaacaWLjaWaaeWaaeaacqaIYaGmaiaawIcacaGLPaaaaaa@46D5@

where *l *is an integer number that corresponds to the filter order, and *a*_0_,..., *a*_*l *_are proper coefficients. With regard to the Kaiser filter theory, the design parameter *l *corresponds to the length of the moving window used by the processing algorithm. The smoothing parameter *l *can be tailored to the specific mass spectrometric signals to be analyzed, which might be characterized by different resolutions and background noise levels.

The resulting spectrum was used to sequentially estimate the baseline drift *c *and the non-uniform noise level *σ*. It was divided into 100 signal blocks *v*_1_, ..., *v*_100 _each with a 150 Da width, and the blocks showing peaks were selected on the basis of the *kurtosis *(*fourth-order cumulant*). After centering a generic signal block *v*, the kurtosis *kurt *could be computed as

*kurt*(*v*) = *E*{*v*^4^} - 3(*E*{*v*^2^})^2 ^    (3)

where *E*{•} is the expectation operator [26].

The kurtosis is a measure of whether the data are peaked or flat relative to a normal distribution: for a zero-mean Gaussian random variable, kurtosis is null; for densities peaked at zero, it is positive, and for flatter densities, it is negative.

The categorization of signal blocks containing peaks, accomplished with the condition *kurt *> 1, was necessary because the peaks might alter the estimates of *c *and *σ*. Consequently, the signal blocks containing peaks were disregarded and the remaining blocks were used to create a vector *x *= {*x*_1_, *x*_2_, ..., *x*_*N*_}, containing the *m/z *values corresponding to the central points of the intervals. The baseline drift *c *was reconstructed using a linear interpolation of the vector *y *= {*y*_1_, *y*_2_, ..., *y*_*N*_} with respect to the points defined in *x *and generated from the average values of the signal within the selected blocks. The baseline was subtracted from the spectrum. A similar approach was used to estimate the variable noise level *σ*, by means of the vector *w *= {*w*_1_, *w*_2_, ..., *w*_*N*_}, containing the standard deviations of the signal within the selected blocks. For each *m/z *value of the mass spectrum, the noise level was used to calculate the SNR, defined as the ratio between the estimated signal intensity *s *and the estimated noise intensity *σ*, according to the equation

SNR=r−cσ     (4)
 MathType@MTEF@5@5@+=feaafiart1ev1aaatCvAUfKttLearuWrP9MDH5MBPbIqV92AaeXatLxBI9gBaebbnrfifHhDYfgasaacH8akY=wiFfYdH8Gipec8Eeeu0xXdbba9frFj0=OqFfea0dXdd9vqai=hGuQ8kuc9pgc9s8qqaq=dirpe0xb9q8qiLsFr0=vr0=vr0dc8meaabaqaciaacaGaaeqabaqabeGadaaakeaacqWGtbWucqWGobGtcqWGsbGucqGH9aqpdaWcaaqaaiabdkhaYjabgkHiTiabdogaJbqaaGGaciab=n8aZbaacaWLjaGaaCzcamaabmaabaGaeGinaqdacaGLOaGaayzkaaaaaa@3A79@

Subsequently, the processed spectrum was analyzed for the first step of the peak-picking procedure: if the point intensity was the highest among its nearest ± *f *points, a peak was detected in that position [8]. The number *f *could be varied to best fit the specific resolution of the mass spectrometer; in this study, *f *was set equal to 2, in order to cover a range of 0.5 Da. The second step of the peak-picking procedure was the elimination of the detected peaks with a SNR lower than a preset threshold. In accordance with the IUPAC guidelines [14], the limit of detection was set to 3σ, corresponding to SNR = 3.

### Separation of protein signals from noise

We compared multiple spectra to measure the reliability of the peaks detected in each single spectrum. However, since the mass spectra could be incompletely aligned after the calibration procedure, a maximum tolerance distance *d *equal to 300 ppm of the *m/z *value was accepted for the comparison [15,16]. The information about the peak intensities was arranged in a [*a *× *b*]-dimensional matrix *R*, where *a *is the number of spectra and *b *the total number of peaks detected in the mass spectra. Each element *r*_*ij *_was set to zero if the *i*-th spectrum did not contain the *j*-th peak, or otherwise set to the related peak intensity. A *b*-dimensional vector *z *containing the *m/z *values corresponding to the peaks was created. The vector *z *and the columns of the matrix *R *were then sorted in ascending order on the basis of the *m/z *value. The matrix dimension was reduced by replacing the columns of *R *for which the elements of *z *were within the distance *d *with a unique column containing their sum. In a similar manner, the same values of *z *within the distance *d *were substituted with their average. Several peak classes were created: a peak detection rate (PDR) was defined as the ratio between the number of spectra presenting the considered peak and the total number of analyzed spectra for each class. The peak classes with a PDR larger than a minimum acceptable PDR level (generally set between 0.4 and 0.6) were arbitrarily assumed to be reliable estimates of the protein signals. When the number of detection errors was relatively low, this criterion allowed us to characterize the "true" peaks in the mass spectrum and to classify those remaining as noise peaks. The selected peak intensities can be used to generate an average mass spectrum, representative of the whole group of analyzed MALDI-TOF mass spectra. When the quantitative analysis of protein peak intensities was required, as for example in case of biomarker discovery, a further selection of the "true" peaks should be performed: following the IUPAC recommendations [14], only the peaks in the average mass spectrum with SNR > 10 were retained.

## Authors' contributions

DM designed and implemented the method, and wrote the first draft of the manuscript. FP assisted with the design of the method, carried out the data analysis, visualized the results and wrote essential parts of the manuscript. DP and PDB performed sample preparations and MS experiments. MDN, CDI and GF were involved in and contributed to the approach used in the data analysis. SC contributed to the discussion of signal processing and manuscript writing. AU and PS supervised the project and provided design oversight. All authors read and approved the manuscript.

## Supplementary Material

Additional File 1 Compressed archive containing the LIMPIC source code, implemented in MATLAB. Click here for file

Additional File 2 Compressed archive of 30 text files, containing MALDI-TOF-MS data acquired from in-vitro protein mixtures. The text files are arranged in 6 folders, each referring to a different cytochrome C concentration. Click here for file

Additional File 3 Compressed archive of 30 text files, containing MALDI-TOF-MS data acquired from human plasma samples. Click here for file

Additional File 4 Spreadsheet of the peak positions and intensities detected with LIMPIC from the MALDI-TOF-MS data referring to human plasma samples. Click here for file

Additional File 5 List of the proteins used for the comparison of LIMPIC, APEX, CENTROID and CROMWELL performances. They correspond to a subset of the proteins that are classified in the Human Plasma Proteome Project (HPPP) database and can be revealed by MALDI-TOF mass spectrometry in the m/z range 5–20 kDa. Click here for file
