# Supplementary material for: LIMPIC: a computational method for the separation of protein MALDI-TOF-MS signals from noise
Source: BMC Bioinformatics. 2007 Mar 26;8:101. doi: 10.1186/1471-2105-8-101 (PMC1847688; doi:10.1186/1471-2105-8-101)
Supplement: Additional File 5 — List of the proteins used for the comparison of LIMPIC, APEX, CENTROID and CROMWELL performances. They correspond to a subset of the proteins that are classified in the Human Plasma Proteome Project (HPPP) database and can be revealed by MALDI-TOF mass spectrometry in the m/z range 5–20 kDa. [file 1471-2105-8-101-S5.pdf]

**Protein list**

Albumin
Alpha-1 Acid Glycoprotein (AGP)
Alpha-1 Antichymotrypsin (ACT)
Alpha-1 Antitrypsin (A1AT)
Alpha-1 Microglobulin (A1M)
Alpha1-Proteinase inhibitor
Alpha-2 Macroglobulin (A2M)
Alpha-Feto Protein (AFP)
Antithrombin III (AT3)
Apolipoprotein A1
Apolipoprotein A2
Apolipoprotein B100
Apolipoprotein C1
Apolipoprotein C1
Apolipoprotein C2
Apolipoprotein C3
Apolipoprotein C3
Apolipoprotein C3
Apolipoprotein C3
Beta-2 Glycoprotein 1 (B2GP1)
Beta-2 Microglobulin (B2M)
Beta-HCG
Beta Trace
C-Reactive Protein (CRP)
Chorionic Gonadotrophin (HCG)
Complement C1q
Complement C3
Complement C4
Creatine Kinase MB (CK-MB)
Creatine Kinase MM (CK-MM)
Cystatin C
Fatty Acid Binding Protein (FABP)
Ferritin
Fibronectin
Follicle Stimulating Hormone (FSH)
Hemoglobin alpha-chain
Hemoglobin beta-chain
Haptoglobin
Hemopexin
Human Placental Lactogen (HPL)
IgA
IgE
IgG
IgM
Immunoglobulin M Kappa
Jo 1
Kappa Light Chain
Lysozyme

Myoglobin
Neurone Specific Enolase (NSE)
Pepsinogen I
Prealbumin
Prolactin
Prostate Acid Phosphatase (PAP)
Prostate Specific Antigen (PSA)
Retinol Binding Protein (RBP)
Serum Amyloid A (SAA)
Sex Hormone Binding Globulin (SHBG)
SS-A Antigen
SS-B Antigen
Tamm Horsfall Glycoprotein
Thyroxine Binding Globulin (TBG)
Thyroglobulin (Tg)
Thyroid Stimulating Hormone (TSH)
Transferrin
Transferrin Receptor (TfR)
Transthyretin
Troponin
Urine Trypsin Inhibitor (UTI)
